# Dual Tasking during Trip Recovery and Obstacle Clearance among Young, Healthy Adults in Human Factors Research

**DOI:** 10.3390/ijerph181910144

**Published:** 2021-09-27

**Authors:** Sachini N. K. Kodithuwakku Arachchige, Harish Chander, Adam C. Knight, Reuben F. Burch V, Chih-Chia Chen, Jennifer C. Reneker

**Affiliations:** 1 Neuromechanics Laboratory, Department of Kinesiology, Mississippi State University, Starkville, MS 39762, USA; hchander@colled.msstate.edu (H.C.); aknight@colled.msstate.edu (A.C.K.); 2 Human Factors & Athlete Engineering, Center for Advanced Vehicular Systems, Mississippi State University, Starkville, MS 39759, USA; burch@ise.msstate.edu; 3 Department of Industrial & Systems Engineering, Mississippi State University, Starkville, MS 39762, USA; 4 Cognitive and Motor Control Laboratory, Department of Kinesiology, Mississippi State University, Starkville, MS 39762, USA; chih.chia.chen@msstate.edu; 5 Department of Population Health Sciences, School of Population Health, University of Mississippi Medical Center, Jackson, MS 39216, USA; jreneker@umc.edu

**Keywords:** ergonomics, falls, cognitive, motor, secondary task, dual task, attention

## Abstract

Trip-induced falls are extremely common in ergonomic settings. Such situations can lead to fatal or non-fatal injuries, affecting the workers’ quality of life and earning capacity. Dual tasking (DT) is a leading cause of trips and ineffective obstacle clearance among workers. DT increases their attentional demand, challenging both postural control and concurrent secondary tasks. As the human brain has limited attentional processing capacity, even young, healthy adults need to prioritize duties during DT. This article aimed to analyze these secondary task types and their applications in recent trip-related studies conducted on young, healthy adults. An extensive review of the recent trip-related literature was performed to provide a condensed summary of the dual tasks used. In previous trip-related literature, distinct types of secondary tasks were used. The choice of the concurrent task must be made vigilantly depending on the occupation, environmental context, available resources, and feasibility. DT can be used as a tool to train workers on selective attention, which is a lifesaving skill in ergonomic settings, especially in the occupations of roofers, construction workers, or truck drivers. Such training can result in successful obstacle clearance and trip recovery skills, which eventually minimizes the number of falls at the workplace.

## 1. Introduction

According to the Bureau of Labor Statistics, in the year 2018, 791 fatal and 240,160 non-fatal injuries were reported due to slips, trips, and falls in ergonomic settings [1]. Such injuries affect the workers’ health and earning capacity while decreasing workplace productivity. A trip is a sudden forward perturbation of the body due to inefficient toe-ground clearance upon contacting an obstacle [2]. Hence, the individual can experience a temporary loss of balance, which can be regained or progress to a fall, depending on the perturbation magnitude. Trip hazards are widespread in ergonomic settings, such as but not limited to steps, ridges, cords, carpets/mats, furniture, tools, and equipment. Further, anterior load carrying, poor lighting, cluttered environments, and high workload make the occupational environments more trip prone. Thus, as a fall prevention intervention, ergonomists are concerned with minimizing trips in the workplace.

Recovery following a postural perturbation such as a trip or successfully crossing an obstacle depends on multiple factors. In both situations, one’s center of gravity (COG) may shift outside the base of support, but in a more voluntary manner in the latter. As this COG shift and suddenly acquired unilateral stance threatens their balance, stability must be regained, for which a robust postural control system is required [3]. Although trip recovery was initially thought of as a reflex-driven process, growing evidence shows an involvement of higher cortical structures [4]. Lack of attention or divided attention is identified as a significant reason for trips and poor trip recovery [5,6]. The attentional requirement for a certain task depends on individual factors, the task at hand, and the surrounding environmental factors. Less attentional resources are needed for simpler tasks such as quiet standing on bilateral feet, while more attentional resources are required during dynamic tasks, perturbations, and alternating sensory outputs [7]. As postural tasks such as walking or obstacle avoidance require considerable attention, an added secondary task causes divided attention in the worker. Moreover, attentional demands are incredibly high at workplaces, depending on the nature of the occupation. Workers are required to perform multiple tasks at once, which can be job-related or general tasks such as walking or speaking. Such multiple tasks can be only physical (motor), only mental (cognitive), or both. As a trip or obstacle clearance increases the demands of the postural control system to counteract the destabilizing forces, an added concurrent task claims more cognitive resources, including divided attention and increasing the risk of tripping [8]. As the human brain has limited attentional resources, some tasks can be unintentionally prioritized once the demands increase, while some are easily neglected. This trade-off occurs according to the attentional requirement of each task and the sensory-motor processing capability of the individual [9].

The dual-task paradigm is a widely used method to assess attentional capacities in many research fields. In the DT paradigm, postural control is considered the primary task and the added secondary performance (e.g., load-carrying) as the secondary task. The speed and accuracy of both task performances are usually affected in DT compared with single tasking [10]. Besides the failures in achieving tasks successfully, DT usually does not cause detrimental effects during day-to-day activities. However, the ergonomic population needs to perform mentally and physically demanding tasks constantly. These workers may have to perform DT while walking (e.g., firefighters, military personnel, roofers) or during quiet stance (e.g., roofers, construction workers). In such populations, a minimal performance decrement in either task can cause catastrophic outcomes (e.g., falling from a height). Therefore, the ergonomic population must be well-trained in performing DT under various circumstances (e.g., poor lighting conditions and rain). While performing a secondary task in trip-related studies, the trip incidence and outcome are shown to vary depending on the nature of the added task [5]. Furthermore, young, healthy individuals perform better during DT than geriatric and clinical populations due to superior physical, physiological, and cognitive abilities [11]. Nevertheless, previous studies on young, healthy adults’ trip recovery and obstacle clearance with dual tasking (DT) is limited. Young adults comprise a significant proportion of the working population, and thus, must be a major demographic group studied in ergonomic fall prevention research. Furthermore, the conclusions of the existing DT and trip-related research in young adults are a bit controversial. Thus, there is always a need for more research to bridge the gap to understand the interference of DT and enhance the performance during DT. As such, a targeted review of such research is much warranted and attempted with this review. There are distinct types of DT incorporated in previous trip recovery and obstacle clearance literature. However, the use, efficiency, feasibility, and acceptance of those secondary tasks are debatable. Therefore, the purpose of this article is to analyze these secondary task types and their applications in recent trip-related studies on young, healthy adults.

**Scheme 1:**
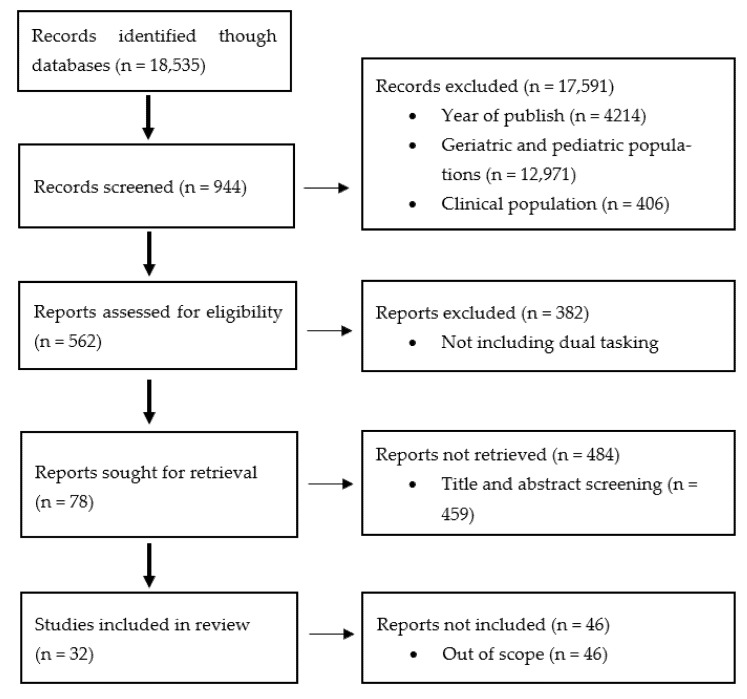
PRISMA flow diagram of the article selection [12].

The journal articles for this review article were selected by a comprehensive literature search performed through Google Scholar and Scopus search engines (Scheme 1). The initial search of the words “trip”, “obstacle”, and “stumble” returned 18535 results. From these, the articles that were published within the previous 20 years (from 2002 to 2021) were selected for screening. Exclusion of the studies with the words, “elderly”, “senior”, “geriatric”, “children”, “pediatric” and “adolescent” yielded 1350 articles, which were further refined with the words “stroke”, “Parkinson”, “TBI (traumatic brain injury)”, “MS (multiple sclerosis)”, “sclerosis”, “prosthetic or prosthesis”, and “amputation or amputee” yielding 944 records. From the resulted number of records, the elimination of the articles with the words “vehicle”, “automobile”, and “robot” and articles without the words “dual”, “attention”, “concurrent”, “simultaneous”, “multi”, or “task” returned 562 results. Screening of the article titles yielded 221 records, which was further refined to 103 articles upon reading the abstract. However, only 78 studies had full-text availability through the authors’ affiliated institutions. Of the selected 78 studies, 46 studies were out of scope, or the outcome was not relevant for the purpose of this study. Thus, 32 articles were included in this literature review. Those incorporated secondary tasks are categorized as cognitive and motor tasks and are described below.

## 2. Secondary Cognitive Tasks

An added task that requires additional mental processing can be categorized as a secondary cognitive task. Identifying, observing, listening, counting, speaking, memorizing, and mentally calculating were used as cognitive tasks in the previous literature (Table 1). While some of these tasks are simple (e.g., the congruent visual Stroop test), some tasks are challenging (e.g., n-back tests). Thus, the selection of the task must be made considering the capabilities of the population.

### 2.1. Vision-Based Cognitive Tasks

Vision-based cognitive tasks are commonly used in trip-related studies. The visual Stroop test [13,14,18] visuospatial attention test [14,15], and visuomotor tracking test [16,17] are some widely used tasks. The visual Stroop test can be congruent (name of the color and the font color are the same) or incongruent (name of the color and the font color are different), and the participants must mention the font color, not the word written [18]. In the visual attention task (VAT), a rectangular white image with three orange “C’s” and one red “C” is projected onto the floor. The participants must identify the opening direction of red “C” [14]. In the visuomotor tracking task (VTT), the participants must continuously track a pseudorandom target displayed on a screen using a pursuit cursor or joystick [16,17]. With a concurrent visual task, increased toe-clearance height, slower foot velocity, longer non-dominant leg stance during obstacle crossing, greater tripping incidences, and increased near falls are reported [13,14,18]. Foot placement during trip recovery or obstacle clearance needs visual information. It is hypothesized that this information is gathered prior to the perturbation and later integrated with the concurrent online information as the perturbation occurs [17]. Visual tasks, trip recovery, and obstacle clearance demand significant attention; thus, competition for attention occurs during DT. Attention requirements for each visual task vary according to the nature of the task [15]. The Stroop test requires direct attention for a longer duration compared with VAT, which requires selective attention for a shorter duration. Due to this difference, the performances are more affected with the Stroop test than VAT [14]. Moreover, vision-based secondary cognitive tasks assess the structural interference which occurs when both simultaneous tasks share a similar input or output of a system [8].

In ergonomic settings, unexpected visual stimuli are extremely common (e.g., a co-worker or a vehicle crossing the path). Thus, the ability to rapidly switch attentional focus is required. Some occupations, such as the military, require sustained attention while navigating through a field. Furthermore, visual-based tasks can conveniently be carried out in an ergonomic environment. However, in tasks such as the Stroop test or VAT, the individuals’ visual acuity and color-blindness must be considered. This issue was addressed by some groups using a Snellen test and Ishihara test to assess visual acuity and color-blindness, respectively [15]. In addition, the projection distance and height of the visual task must be standardized. Although the image is projected onto the floor in VAT, displaying targets at eye height is favored. Moreover, in computer-based tasks, all workers may not be familiar with using a cursor or a joystick which can hinder their performance.

### 2.2. Auditory Cognitive Tasks

The congruent or incongruent auditory Stroop test is a frequently used auditory cognitive task with young adults [5,8,18,19,20,21,22]. Here, the participants must recognize the pitch (high or low) of the words being said. In the congruent test, the pitch and the cue are matched (the word “low” is spoken in a low pitch), while in the incongruent test, the pitch and the cue are not matched (the word “low” is spoken in a high pitch) [5]. Similar to the visual Stroop test, the auditory test requires greater cognitive processing and inhibitory attention. With auditory-based tasks, reduced obstacle crossing swing velocities, higher stiffness of the crossing limb, and higher toe-clearance heights are reported [5,8,18,19,20,21,22]. Auditory-based cognitive tasks are a better method to assess capacity interference as opposed to the structural interference assessed by vision-based tasks. Capacity interference occurs once two simultaneous tasks overload the general central processing ability. In order to identify the interference of a cognitive task on a task that involves the postural control system, it is more beneficial to include a task that does not cause structural interference [22]. This was well-explained in the studies which compare the effect of both the visual and auditory Stroop tests and showed greater performance decrements with the visual Stroop test [18].

The auditory Stroop test is not very applicable to real life. However, during day-to-day life activities and at the workplace, individuals must listen to things while performing a motor task (e.g., listening to a co-worker while stepping onto a ridge). Auditory tests can be efficiently conducted at the workplace, and if the workplace is noisy, headphones can be used to provide auditory input. As a method to improve the variability of the auditory Stroop test and further challenge the participants, in some studies, both male and female voices are included randomly. However, it can cause some confusion to the participants (e.g., high pitch male voice may mimic a low-pitched female voice) [22].

### 2.3. Speech and Verbal Tasks

Speech is not a commonly used secondary cognitive task with young adults. However, talking while obstacle crossing [23] and talking on the phone while approaching an obstacle has been performed [24]. Speech is a complex cognitive task that requires mental retrieving of information, translating it to words, producing sound, pronouncing the words properly, and correcting the output grammatically. Therefore, it is a challenging cognitive task, although performed very frequently in real life. As speech is more associated with socializing, young adults prioritize speech over the postural task with easier gait tasks; however, both tasks (speech and obstacle clearance) are affected once the task becomes difficult [23].

Performing verbal tasks and obstacle crossing is very common in occupational settings (e.g., talking to a co-worker while maneuvering through a cluttered environment). In conducting speech-related studies, attention must be paid to choose day-to-day topics (e.g., pets, sports, food) depending on the person’s interest. In addition, advising them to “speak as if you are walking to work with a friend” promotes better outcomes [23]. As speech is a more natural task than performing an unfamiliar task (the Stroop test), worker compliance for the study will be higher. Open-ended questions must be used to achieve a continuous flow of words. However, the number of pauses within one trial can vary among the participants.

### 2.4. Memory-Based Cognitive Tasks

Compared with other cognitive tasks, the young adults’ obstacle clearance was minimally affected by this task [9,25]. The digit monitoring test and n-back working memory (WM) test are two identified memory-based tasks in the literature [9,25]. In the digit monitoring test, the experimenter informs the participant of a random number between 1 and 9 before the trial. Upon starting the trial, the participants hear a sequence of numbers from 1 to 9, and they must count the times they hear the target number and report it back to the investigator at the end of the trial [9]. In the n-back WM test, the participants must monitor one verbal signal, and one non-verbal signal presented simultaneously at fixed intervals. The participants must react whenever the current signals are similar to the signals presented at the nth interval. This is a more complex task that needs higher cognitive processing. As the value for “n” increases, the complexity of the task increases, which usually affects the outcome of both tasks [25]. With an added memory-based cognitive task, the resulting shorter step length, slower gait speed, and greater gait variability, caused the obstacle negotiation to be more challenging [25].

These memory-based tasks must be implemented according to the cognition level of the participants and can be performed with minimal resources. Memorizing something or working while keeping something in mind is common in the workplace (e.g., doing inventory). Delivering the auditory commands through headphones are applicable for noisy ergonomic settings. However, some tests, such as the 3-back WM test, are highly challenging and thus may not be suitable for all employee categories.

### 2.5. Other Cognitive Tasks

Secondary tasks such as serial sevens [6] and mental mathematical quizzes [26] are used in young adults’ trip-related studies. In serial sevens, the participants are asked to subtract sevens backward from a given number, while in the alpha-numeric sequencing task, the participants are requested to create an alternating sequence of letters and numbers (e.g., A-1, B-2). Similar to the memory-based tasks, these tasks show minimal effects on young adults [6]. The minimal effects suggest that young adults have better motor-cognitive resources, allowing them to succeed in both tasks. Although performing a concurrent cognitive task is expected in ergonomic settings, the tasks mentioned above can be unfamiliar. These tests require a tremendous amount of cognitive processing and must be administered after analyzing workers’ capabilities. If including repeating serial sevens trials in a study, providing different starting numbers (e.g., starting from 200, 100, and 50) will prevent learning effects [6].

## 3. Secondary Motor Tasks

An added task that requires the participants to perform an additional physical activity can be categorized as a secondary motor task. Object carrying, holding, and manipulating were used as secondary motor tasks in the previous literature (Table 2). Simultaneous motor tasks are widespread in ergonomic settings. In fact, a significant proportion of falls at construction sites are shown to occur during carrying tools, objects, or machinery [27].

### 3.1. Anterior Load Carrying

The young adults’ performance in obstacle clearance is affected by load-carrying and further affected with increasing loads [28]. During load carrying, the added load shifts the combined center of mass of the body and the load anteriorly and upwards, threatening postural stability. This increases the attentional demands than when crossing an obstacle without a load [30]. Further, an anterior load occludes the participants’ vision of the walking path/feet. While this visual occlusion increases the risk of trips, it makes the participants pay additional attention to their proprioception, increasing cognitive demands [32]. Furthermore, navigating through a hazardous area is usually guided by a feedforward mechanism, which becomes impossible due to vision obstruction [33].

Load-carrying can be easily adopted for DT as it requires minimal resources. The load complexity can be changed depending on the participants, even according to their body weight or carrying other types of loads (posterior, lateral, and unstable loads). Anterior and other types of load-carrying are an incredibly common secondary task in ergonomic settings. However, it is uncertain whether the results occur due to attentional demands increased by load-carrying itself or the visual occlusion. Addressing this issue, Jehu et al. (2019) compared carrying an opaque load and a clear load, which showed poor performance with the opaque load, showing the contribution of visual obstruction [29]. However, since both postural control and load carrying involve the musculoskeletal system, it can cause both structural and capacity interference. Further, in a load-carrying task, the results can be contingent upon the participants’ strength; however, it is probably not an issue for young, healthy adults.

### 3.2. Carrying, Holding, or Manipulating Smaller Objects

Holding a weighted tray with an empty cup during treadmill-based trip perturbations [31] and carrying a tray with weighted cups during obstacle clearance [23] has been previously used as secondary tasks. In Sung et al. (2020), the participants were advised to prevent the cups from falling off while recovering from the trip, further increasing the participants’ cognitive demand. Even though the tray was not heavy, the participants’ gait was affected by carrying it compared with clearing the obstacle without a tray [23]. In Raffegeau et al. (2018), when the participants were advised to clear an obstacle while talking and carrying a tray with weighted cups, both speech and obstacle clearance were affected, showing the difficulty of attention allocation [23]. Carrying or holding an object is a common real-life scenario at the workplace; therefore, the participants may perform better than when performing an unfamiliar traditional cognitive task (e.g., the Stroop test). Since the participants have a visible consequence upon failing the task (cups falling off), this secondary task may be more practical than a task with no specific consequence (e.g., VAT). Since keeping the cups on the tray without falling off adds an extra cognitive load, the results can be due to holding the tray, focusing extra attention to maintain the cups on the tray, or both. Further, carrying something in the hands can influence trip outcomes due to the absence of upper extremity movement upon application of the perturbation. The task can be more applicable to the working population by using something relevant to the workplace, such as a tool or a handheld device instead of a tray.

## 4. Combination of Cognitive and Motor Tasks

Some previous studies have required the participants to carry or manipulate an object, such as carrying a mobile phone [24,25,26,34], typing/pressing keys on a mobile phone [24,25,26,34], rotating a potentiometer with a thumb [17], or using a joystick [16] as the method to perform another secondary task. Although these groups only assessed the outcomes with the main secondary cognitive task, these added fine motor tasks could have changed the results. An additional trial without such fine motor tasks (“neutral trial”) can aid in differentiating the outcome.

### Using Mobile Phones

Using a mobile phone can be considered as a combination of motor and cognitive tasks. To perform visual (texting), auditory (listening), verbal (talking), or other cognitive tasks (completing a quiz) using the phone, the participants are required to hold and manipulate the phone. With the increased use of cell phones among young adults, reading or writing text messages [26,34], and talking on the phone [24] have been incorporated in recent studies (Table 3). The authors reported smaller step length during obstacle approaching and crossing, greater toe-obstacle clearance, slower foot movement over the obstacle, and increased medial-lateral deviations during crossing due to the simultaneous mobile phone use [24,34]. The effects were highest with writing text messages, followed by reading text messages, and lowest while talking on the phone. The minimal visual information of the surroundings due to greater head flexion during texting is explained as a possible reason for this order [24]. Further, attentional demands increase during texting due to the frequent reorientation of vision between the phone and the walking path [34].

Mobile phone use is an extremely common real-world task, even in ergonomic settings. It is a convenient secondary task to be added to a study that does not require additional resources. Including simple things for texting and not altering how the participants hold/interact with the phone promotes the natural behavior of the participants [34]. However, familiarity bias was identified as a disadvantage, which some researchers prevented by providing a lab-owned mobile phone to use during the study [26]. In assessing the talking over the phone condition, participants are not allowed to use the speakerphone function but must directly hold the phone next to their ear. By adding eye-tracking technology, attention deviations/prioritizations can be further investigated [24,26].

## 5. Limitations and Future Suggestions

In previous trip-related studies with DT, a few limitations were noticed. Although many researchers intend to observe the impact of one secondary task, sometimes more than one task was recognized. For instance, in Timmis et al. (2017), the participants were required to memorize a received text message and report it back to the investigator, which can be considered a memorization task. In Pitman et al. (2021), a visuomotor task was conducted, which takes away a significant portion of the vision. Further, having to respond verbally can be considered as an additional verbal task, e.g., verbally respond to the visual Stroop test [13,14], or the auditory Stroop test [5,8,19,20,22], or a VAT [14,15]. Therefore, performing a control trial with no secondary tasks, and further DT trails with standardized and normalized intrinsic (human) factors and external (environmental) factors to understand the impact of the secondary DT task on the task of interest must be encouraged.

Furthermore, the studies discussed here were conducted in research labs or under controlled conditions. Nevertheless, the real-world scenarios can be highly different (e.g., occupational settings such as a construction and industrial sites with crowded and cluttered walkways, unfamiliar and uneven walking surfaces, or walking at an elevation). Moreover, the nature of the provided instructions to the participants must be well-tailored, and they must not be informed which task to prioritize. For example, “walk as fast as you can” can make the participants prioritize walking, while “respond as quickly and accurately as possible” may lead to prioritizing the cognitive task. In some studies, the participants were told their number of correct responses after each trial (e.g., the Stroop test), which can cause additional anxiety to them or an intent to perform differently in the next trial, which can impact observed results. However, instructions to the participants should be catered to the research question. For example, if the research question is to assess motor learning with the presence of verbal feedback, then the above instructions would be ideal. Further, in conducting these attention-demanding studies at the workplace, the workers’ mental and physical fatigue levels must be considered before starting data collection [5]. Allowing practice trials are essential with tasks such as the n-back test, but excessive practice can cause a learning effect. In addition, randomizing obstacle location, normalizing the obstacle to subject height [17,18], and keeping the perturbation magnitude constant can elicit better outcomes. Upon minimizing these limitations, the DT paradigm can be a great approach to understand the attention allocation among young adults.

## 6. Conclusions

The secondary tasks that were used in the previous literature have their unique advantages and disadvantages. For instance, vision-based DT is extremely common in ergonomic settings (e.g., identifying safety hazards); thus, adding a vision-based task is more applicable. However, the occupational tasks that are similar to the incongruent visual Stroop tests may not be highly prevalent in these settings. Similarly, simultaneous auditory tasks are widespread among workers (e.g., following verbal instructions while working), but tasks similar to the auditory Stroop test are rare. Moreover, adding a mental math task as the secondary task would be convenient for the investigator; however, the employee’s education level can possibly affect the results. While anterior load carrying is a common motor DT in ergonomic settings, studying the participants during lightweight object carrying can be undervaluing. Hence, the choice and application of the secondary tasks must be made vigilantly in DT-related studies, considering the nature of the occupation of interest. Furthermore, DT can be an excellent tool to train workers on attention switching, which is a lifesaving skill in ergonomic settings, especially in the occupations of roofers, construction workers, or truck drivers. Such trained workers can acquire successful obstacle avoidance and trip recovery skills, which aids in minimizing the number of falls at the workplace.

## Figures and Tables

**Table 1:** Secondary cognitive tasks during trip recovery and obstacle clearance.

Article	Perturbation Method	Primary Task	Secondary Cognitive Task
Vision-based cognitive tasks			
Kim, 2008 [13]	Obstacle crossing	Walking	Visual Stroop task
Lo and Chou, 2015 [14]	Obstacle crossing	Walking	Visuospatial attention task Visual Stroop task
Lo et al., 2015 [15]	Obstacle clearance	Walking and standing	Visuospatial attention task
Quant et al., 2004 [16]	Translation of a moving platform	Standing	Visuomotor tracking task
Zettel et al., 2005 [17]	Backward platform perturbation	Standing	Visuomotor tracking task
Visual and auditory cognitive tasks			
Worden et al., 2016 [18]	Obstacle crossing	Walking	Visual Stroop task Auditory Stroop task
Auditory cognitive tasks			
Inkol et al., 2018 [5]	Backward platform perturbation	Standing	Auditory Stroop task
Pitman et al., 2021 [19]	Obstacle crossing	Walking	Auditory Stroop test
Siu et al., 2008 [20]	Obstacle avoidance	Walking and sitting	Auditory Stroop task
Weerdesteyn et al., 2003 [8]	Obstacle clearance	Walking	Auditory Stroop task
Worden et al., 2016 [21]	Obstacle avoidance	Walking and standing	Auditory Stroop task
Worden and Vallis, 2014 [22]	Obstacle avoidance	Walking	Auditory Stroop task
Speech and verbal tasks			
Raffegeau et al., 2018 [23]	Obstacle crossing	Walking	Speech
Timmis et al., 2017 [24]	Obstacle negotiation	Walking	Talking on the mobile phone
Memory-based cognitive tasks			
da Silva Costa et al., 2018 [9]	Obstacle avoidance	Walking	Digit-monitoring test
Lin and Lin, 2016 [25]	Obstacle crossing	Walking	60-s 1-back and 3-back working memory tasks
Other cognitive tasks			
Licence et al., 2015 [26]	Obstacle avoidance	Walking	Responding to text messages Completing a mental mathematics quiz on the phone
Paran et al., 2020 [6]	Treadmill-induced trip	Treadmill walking	Serial subtractions

**Table 2:** Secondary motor tasks during trip recovery and obstacle clearance.

Article	Perturbation Method	Primary Task	Secondary Motor Task
Anterior load carrying			
Hawkins et al., 2011 [28]	Obstacle crossing	Walking	Anterior load carrying
Jehu et al., 2019 [29]	Obstacle clearance	Walking	Anterior load carrying
Rietdyk et al., 2005 [30]	Obstacle negotiation	Walking	Anterior load carrying
Carrying, holding, or manipulating smaller objects			
Raffegeau et al., 2018 [23]	Obstacle crossing	Walking	Carrying a tray and cups
Sung et al., 2020 [31]	Treadmill-induced trip	Treadmill walking	Holding a weighted tray with an empty cup

**Table 3:** Secondary cognitive and motor task combinations during trip recovery and obstacle clearance.

Article	Perturbation Method	Primary Task	Secondary Cognitive and Motor Tasks
Chen et al., 2018 [34]	Obstacle crossing	Walking	Responding to a text message and carrying a mobile phone
Licence et al., 2015 [26]	Obstacle avoidance	Walking	Responding to standardized texting questions, completing a mental mathematics quiz, andcarrying a mobile phone
Timmis et al., 2017 [24]	Obstacle negotiation	Walking	Talking on the phone, read a text message, write and send a text message, and carrying a mobile phone

## Data Availability

Not applicable.
